# The Ethics of Operations

**Published:** 1896-03-07

**Authors:** 


### The Hospital

An Institutional Journal of

The Medical Sciences and Hospital Administration, with

Vol. XIX.—No. 493.] AN EXTRA NURSING SUPPLEMENT. [March 7, 1896.

### The Ethics of Operations.

There are several distinct classes of persons and interests involved in the case of surgical operations, namely, patients, their friends, doctors, nurses, fees, incidental expenses, and the feelings and instincts of the community as a whole. Medicine is becoming not merely a highly scientific, but also a highly moral calling, and many of its professors are regarded as men of "light and leading," not in their own art alone, but in all sorts of general questions which go to make up what may be called the "humanitarianism" of the times. We are now, by virtue of our science and the general esteem in which we are held, as "cities set on hills," and our doings and the principles by which we regulate them can by no means "be hid."

A detailed history of the surgical operations, say, of the past fifty years, with the facts that led up to them, the circumstances which attended them, and the uncoloured motives of the surgeons who made and who assisted at the making of them, would afford much food for reflection to the student of human nature. Some motives and circumstances would no doubt be revealed by such a history which would look anything but pleasing in the clear light of reason and of the principle of "doing as one would be done by." But the experience of the average medical man will bear out this general contention, that taking into consideration the absolutely untrammelled and free position in which the surgeon finds himself who is called upon to decide whether a serious operation shall or shall not be performed, and the financial interest he has in performing rather than in advising against operations, the total number of unjustifiable operations—that is, operations performed in the interests of the attendant surgeons rather than in those of the patient—would be found to be exceedingly small. The dangerous sickness of a fellow-creature, and the trust which he is compelled perforce to place in his medical attendants, exalt even the poorest in conscience and the lowest in benevolence of those attendants to an elevation of pure sympathy and of right-minded goodwill which amounts almost to moral heroism. It is human nature itself, and human nature at its very sublimest and best, which stands between the helpless sick and any low and sordid motives which some, even of surgeons, may have in common with the less worthy of their fellows of other callings.

But these are days of microscopic criticism and "interior photography" in more senses than one. There are no questions of general interest to mankind which can be kept entirely secret from mankind. Doctors and their ways are of the deepest interest to the civilised man in all his stages, from the cradle to the grave, and there is a suspicion abroad that surgical operations, taken generally, are not always decided upon from the one and only standpoint of each particular patient's interest and well-being. Such suggestions and suspicions are unfortunately by no means uncommon. They involve the honour of the medical profession and the trust and happiness of the public. There are those who tell us there is no way out of such difficulties, and that the public, when it is helplessly sick, is obliged to trust us, whether it will or not, and that, therefore, we are masters of the situation. Happily that is not so. There is a way out of such difficulties, and a very excellent one. Of late years there has been found among us an increasing class of what may be called "judicial" as against mere "operating surgeons." These are surgeons whose age is such that they no longer care for the worry of operations, but whose mental faculties are still unimpaired, whose experience of "cases" and of the results of all classes of operation is of the widest, and whose judgment is of the ripest and best. These gentlemen, no longer wishing to operate themselves, may very properly be consulted as to whether an operation is or is not desirable. If one such judicial surgeon decides in favour of operation, then an operator can be selected after the decision has been made. There are, in short, "counsellors" and "judges in the medical land"; and the family practitioner and his patient would be well advised in all cases in private practice, where a serious operation is at issue, to take the opinion of such "counsel" and "judges" before entering upon the life and death struggle of the anæsthetic and the operation table. We take the opportunity here of expressing the general opinion that, however it may become a matter of familiarity and routine to the surgeon, an operation cannot ever be anything but a question of profound importance to the patient, and that the only thing which can justify the unconsciousness and danger of an anæsthetic, with the suffering and risk of an operation, is the dangerous necessity of the patient himself. Even in hospitals where the common rule is to have consultations before major operations are performed, such operations should never be undertaken except with the full and mature consent of the patient himself and of his friends.

